# Structural determinants specific for retromer protein sorting nexin 5 in regulating subcellular retrograde membrane trafficking

**DOI:** 10.7555/JBR.37.20230112

**Published:** 2023-11-15

**Authors:** Qing Chen, Meiheng Sun, Xu Han, Hongfei Xu, Yongjian Liu

**Affiliations:** 1 Jiangsu Key Laboratory of Xenotransplantation, and Department of Medical Genetics, Nanjing Medical University, Nanjing, Jiangsu 211166, China; 2 Department of Neuroscience, University of Pittsburgh Kenneth P. Dietrich School of Arts and Sciences, Pittsburgh, PA 15260, USA

**Keywords:** vesicular monoamine transporter 2, SNX5, SNX6, retromer, retrograde trafficking

## Abstract

The endosomal trafficking of signaling membrane proteins, such as receptors, transporters and channels, is mediated by the retromer-mediated sorting machinery, composed of a cargo-selective vacuolar protein sorting trimer and a membrane-deforming subunit of sorting nexin proteins. Recent studies have shown that the isoforms, sorting nexin 5 (SNX5) and SNX6, have played distinctive regulatory roles in retrograde membrane trafficking. However, the molecular insight determined functional differences within the proteins remains unclear. We reported that SNX5 and SNX6 had distinct binding affinity to the cargo protein vesicular monoamine transporter 2 (VMAT2). SNX5, but not SNX6, specifically interacted with VMAT2 through the Phox domain, which contains an alpha-helix binding motif. Using chimeric mutagenesis, we identified that several key residues within this domain were unique in SNX5, but not SNX6, and played an auxiliary role in its binding to VMAT2. Importantly, we generated a set of mutant SNX6, in which the corresponding key residues were mutated to those in SNX5. In addition to the gain in binding affinity to VMAT2, their overexpression functionally rescued the altered retrograde trafficking of VMAT2 induced by siRNA-mediated depletion of *SNX5*. These data strongly suggest that SNX5 and SNX6 have different functions in retrograde membrane trafficking, which is determined by the different structural elements within the Phox domain of two proteins. Our work provides a new information on the role of SNX5 and SNX6 in the molecular regulation of retrograde membrane trafficking and vesicular membrane targeting in monoamine neurotransmission and neurological diseases.

## Introduction

The subcellular endosomal trafficking of membrane proteins is highly regulated by retromer-mediated sorting machinery that is composed of a cargo-selective vacuolar protein sorting (VPS) trimer and a membrane-deforming subunit of sorting nexin (SNX) proteins ^[ 1– 2] ^. While VPS trimer (Vps26/29/30) has been considered as the cargo-selective complex (CSC) ^[ 3] ^, VPS35 is believed to play a role in recruiting cargo proteins, such as the cation-independent mannose-6-phosphate receptor (CI-MPR) for retrograde trafficking from the endosome to the trans-Golgi network (TGN) ^[ 4] ^. Importantly, sorting nexin subunits, including SNX1/2 and SNX5/6, have recently been indicated to be critical for initiating the sorting and targeting of cargo proteins to TGN membranes ^[ 3, 5– 6] ^. Although SNX5 and SNX6 share a 79% similarity in their amino acid sequences and they function as ''twins'' to sense changes in membrane curvature ^[ 7] ^, they are reported to be functionally distinct in several key steps. First, SNX5 and SNX6 differ in the binding spectra of phosphoinositides. In addition to its weak binding to PI(3)P ^[ 8– 10] ^, SNX5 was shown to specifically bind to PI(4,5)P _2_ according to nuclear magnetic resonance structure analysis, suggesting its involvement in the process of cargo protein transport at the site of the plasma membrane; on the other hand, SNX6 was indicated to interact with PI(4)P, which was enriched in the Golgi apparatus ^[ 11] ^. Second, SNX5 and SNX6 may also differ in their interaction with different sorting machineries. SNX5 was reported to bind to the cytoskeleton protein DOCK180, thereby regulating the retrograde transport of CI-MPR ^[ 12] ^, but SNX6 interacted with p150 ^Glued^ in the dynein-dynein activator protein complex, indicating a role in the cargo protein transport along the microtubules and accurately unloading at the TGN ^[ 13– 14] ^. Notably, SNX5 has been reported as the only sorting protein located in synaptic vesicles among the 33 known members of the sorting protein family ^[ 15] ^. Importantly, our recent results also showed that SNX5, but not SNX6, interacted with the vesicular monoamine transporter 2 (VMAT2). However, the structural determinants essential for their functional differences have not been elucidated.

In the current study, we used both CI-MPR and VMAT2 as cargo proteins to biochemically and functionally confirm their distinct interactions with SNX5 and SNX6, respectively. Taking advantage of the amino acid sequence differences between the two SNXs, we then used a chimeric mutagenesis approach to identify that the Phox (PX) domain of SNX5 (91–140) interacted with VMAT2, which could be inhibited by the sequences from a third of either N- or C-terminus of the SNX6 PX domain. Through point mutagenesis, we further identified individual residues unique to SNX5 that were required for the SNX5 interaction with VMAT2. These results provided ample evidence that SNX5 and SNX6 had distinct functions and molecular mechanisms in cargo interaction. Furthermore, our work provides a new experimental basis and direction to investigate the molecular mechanism of retromer components in regulating retrograde membrane trafficking.

## Methods and materials

### Cell culture and transfection

All cells used in the current study were cultured in a 37 ℃ incubator with 5% CO _2_. HeLa and COS-7 cells were maintained in high glucose Dulbecco's Modified Eagle Medium (DMEM, Gibco, Carlsbad, USA) supplemented with 10% fetal bovine serum (FBS, HyClone, Logan, USA) and 1% penicillin/streptomycin (Gibco). PC12 cells were maintained in DMEM supplemented with 10% defined equine serum (DES, HyClone), 5% FBS, and 1% penicillin/streptomycin. Stable lines of rat 3Flag-VMAT2 were prepared as previously described ^[ 16] ^.

Plasmid constructs and siRNA transfection were performed using Lipofectamine 2000 (Invitrogen, Carlsbad, USA) according to the manufacturer's protocol. The transfected cells were harvested and analyzed 24 to 48 h later.

### Plasmid constructions and mutagenesis

The 3Flag-TacM plasmid was constructed as previously described ^[ 17] ^. 3HA-SNX5, 3HA-SNX6, and 3HA-VMAT2 were subcloned into the pcDNA3.1-3HA vector using *Kpn* Ⅰ and *Not* Ⅰ multicloning sites. Plasmids for a series of chimeric proteins, such as 3HA-SNX6 ^5A^, 3HA-SNX6 ^5B,^ 3HA-SNX6 ^5C^, 3HA-SNX6 ^A+BAR^, 3HA-SNX6 ^B+BAR^, 3HA-SNX6 ^C+BAR^, 3HA-SNX5 ^B+BAR^, 3HA-SNX6 ^5A1^, 3HA-SNX6 ^5A2^, 3HA-SNX6 ^5A3^, 3HA-SNX6 ^5C1^, and 3HA-SNX6 ^5C2^, were constructed using PCR to amplify the cDNA inserts followed by their subcloning into pcDNA3.1-3HA vector in the lab using *Kpn* Ⅰ and *Not* Ⅰ multicloning sites. The series of point mutants, such as 3HA-SNX5 (Y132D), 3HA-SNX5 (F136D), 3HA-SNX6 (A30P) ^PX^, 3HA-SNX6 (S37P) ^PX^, 3HA-SNX6(N62P) ^PX^, 3HA-SNX6 (M143S) ^PX^, 3HA-SNX6 (C149Q) ^PX^, 3HA-SNX6 (R158S) ^PX^, 3HA-SNX6(L161R) ^PX^, and 3HA-SNX6 ^tetra-Mut^, were generated by overlay site-directed mutagenesis for generating inserts followed by their subcloning into pcDNA3.1-3HA. Similarly, the constructs of His-recombinant proteins, such as 6His-SNX5 ^PX^, 6His-SNX6 ^PX^, 6His-SNX5-A ^5^, 6His-SNX5-B ^5^, 6His-SNX5-C ^5^, 6His-SNX6-A ^6^, 6His-SNX6-B ^6^, and 6His-SNX6-C ^6^, were subcloned into pET28a(+) vector using *Nde* Ⅰ and *Bam*H Ⅰ multicloning sites. All constructions were confirmed by DNA sequencing.

### Antibodies and siRNA

The primary antibodies used in the study were rabbit polyclonal anti-HA (1∶2000 dilution; Cat. #923501, Biolegend, San Diego, USA), mouse monoclonal anti-HA.11 (1∶2000 dilution; Cat. #901513, Biolegend), rabbit polyclonal anti-Flag (1∶2000 dilution; Cat. #F7425, Sigma-Aldrich, St. Louis, Missouri, USA), mouse monoclonal anti-Flag M2 (1∶2000 dilution; Cat. #F3165, Sigma-Aldrich), rabbit polyclonal anti-secretogranin Ⅱ (SgⅡ) (1∶1000 dilution for Western blotting, 1∶300 for immunofluorescence; Cat. #20357-1-AP, Proteintech, Chicago, USA), sheep polyclonal anti-TGN46 (1∶300 dilution; Cat. #AHP500, AbD Serotec, Kidlington, UK), mouse monoclonal anti-SNX5 (F-11) (1∶500 dilution; Cat. #sc-515215, Santa Cruz, Texas, USA), mouse monoclonal anti-SNX6 (D-5) (1∶500 dilution; Cat. #sc-365965, Santa Cruz), mouse monoclonal anti-β-actin (1∶5000 dilution; Cat. #66009, Proteintech), and mouse monoclonal anti-GAPDH (1∶5000 dilution; Cat. #60004, Proteintech). Secondary antibodies used were goat anti-mouse IgG (H+L) (1∶2000 dilution; Cat. #115-035-003) and goat anti-rabbit IgG (H+L) (1∶2000 dilution; Cat. #111-035-003) conjugated to HRP (Jackson, Pennsylvania, USA) as well as Alexa Fluor 488-conjugated goat anti-mouse IgG (H+L) (Cat. #A-11001), Alexa Fluor 568-conjugated goat anti-rabbit IgG (H+L) (Cat. #A-11011), and Alexa Fluor 488-conjugated donkey anti-sheep IgG (H+L) (Cat. #A-11015, Thermo Fisher Scientific, Massachusetts, USA) that were diluted in 1∶300.

The siRNA oligonucleotides and corresponding scramble siRNA were obtained from GenePharma (Shanghai, China) and resuspended in double-distilled water according to the manufacturer's instructions. Sequences used for human *SNX5* siRNA interference were 5′-GCUGCUAAGGAUCUCUUAUTT-3′ and 5′-GCUUACAUAGCCUGGCUUUTT-3′. The sequences for human *SNX6* were 5′-GGAACUGGCAGAGUUAGAATT-3′. The sequences used for rat *Snx5* were 5′-GUGGCAGCAUUUCGAAAGATT-3′ and 5′-GCUGCAUUGAUUUAUUCAATT-3′. The sequences used for the rat *Snx6* were 5′-CAGGACUCCACAGAUAUAUTT-3′ and 5′-GGCUUCAUGAUUCCUUUGUTT-3′.

### Western blotting

Western blotting was performed as previously described ^[ 18] ^. Briefly, equal amounts of protein samples in sample buffer (Tris-HCl, pH 6.8, 30% glycerol, 10% SDS, 0.6 mmol/L DTT, and 0.012% bromophenol blue) were separated by electrophoresis through discontinuous 10% SDS polyacrylamide gels and transferred to a nitrocellulose membrane (Merck Millipore, Saint Charles, USA). The membrane was then blocked in Tris-buffered saline (TBS) containing 0.1% Tween-20 and 5% non-fat dry milk for 30 min at room temperature (RT) and incubated with primary antibody at 4 ℃ overnight. The next day, the membrane was washed in TBST and incubated in an appropriate secondary antibody conjugated to HRP, followed by washing in TBST and visualization by an enhanced chemiluminescence with the Tanon 5200 gel imaging system (Tanon, Shanghai, China).

### Immunoprecipitation (IP)

The transfected cells were washed with ice-cold phosphate-buffered saline (PBS) once and then lysed in lysis buffer (150 mmol/L NaCl, 50 mmol/L Tris-HCl, pH 8.0, 5 mmol/L EDTA, and 0.4% NP-40) containing protease inhibitors on ice. Cell debris was centrifuged at 15000 *g* for 5 min, and the supernatants were collected and incubated with antibodies for 2 h at 4 ℃ followed by incubation with 30 μL pre-washed protein A/G agarose (Thermo Fisher Scientific) for an additional 2 h. After immunoprecipitation, the beads were washed in lysis buffer three times and then eluted in sample buffer before subjecting to SDS-PAGE analysis.

### Recombinant protein expression and purification

The expression and purification of recombinant proteins were performed as previously described ^[ 19] ^. Briefly, His-tagged SNX5 ^PX^, SNX6 ^PX^, SNX5-A ^5^, SNX5-B ^5^, SNX5-C ^5^, SNX6-A ^6^, SNX6-B ^6^, and SNX6-C ^6^ were produced in *Escherichia coli* BL21 cells using 0.4 mmol/L isopropyl-beta-D-thiogalactopyranoside (IPTG; Biosharp, Beijing, China) for induction at 16 ℃ for overnight. The recovered bacteria were lysed with sonication in lysis buffer (1% Triton X-100 in PBS containing protease inhibitors). The fusion proteins were purified with Ni-NTA agarose (Qiagen, San Francisco, CA, USA). The recombinant proteins were detected by Coomassie brilliant blue.

### His pull-down assay

For the pull-down of Flag-tagged TacM with His-fusion proteins of SNX5, SNX6, and their truncations, cells were transfected with plasmids for overexpression of Flag-TacM. Then, cells were collected and lysed using lysis buffer 24 h after transfection. The clear cell lysates prepared were incubated with equal amounts of His-fusion proteins for two hours at 4 ℃. His-fusion proteins were recovered by incubating with Ni-NTA agarose. The bound proteins on the beads were eluted using 2× sample buffer and separated by SDS-PAGE for immunoblotting using an anti-Flag antibody.

### Equilibrium density gradient fractionation

Stably transformed or transiently transfected PC12 cells were harvested in buffer A (in mmol/L: 150 NaCl, 1 EGTA, 0.1 MgCl _2_, and 10 Hepes, pH 7.4) with a proteinase inhibitor. Cells were homogenized by eight passes through a ball-bearing device (clearance 12 µm). Postnuclear supernatants were then loaded onto continuous density gradients prepared with a gradient mixer using 0.65 mol/L and 1.55 mol/L sucrose in buffer B (in mmol/L: 1 EGTA, 1 MgCl _2_, and 10 Hepes, pH 7.4) and centrifuged in an SW41 rotor (Beckman Instruments, Fullerton, CA, USA) at 153900 *g* for 16 to 18 h at 4 ℃. Fractions (0.5 mL) were collected from top to bottom, and equal amounts of each were denatured in 6× SDS sample buffer and separated and detected by Western blotting.

### Immunofluorescence and confocal microscopy

For cell staining, the process was performed as previously described ^[ 20] ^. In brief, cells were seeded on coverslips coated with poly-D-lysine (PDL, Millipore/Sigma, St. Louis, USA) and Matrigel (BD Biosciences, Franklin Lakes, USA). After being transfected by Lipofectamine 2000 for 24 to 48 h, cells were fixed with 4% PFA (Biosharp) in PBS at RT for 15 min followed by permeabilizing and blocking at RT for 15 min in a blocking solution (2% BSA, 1% fish skin gelatin, and 0.02% saponin in PBS). Cells were then incubated for two hours at RT with primary antibodies, and washed three times in the blocking solution, followed by incubation with appropriate Alexa 488 or 568-conjugated secondary antibodies for two hours. After washing again three times, coverslips were mounted on glass microscope slides using the Fluorescent Mounting Medium (Thermo Fisher Scientific).

For confocal laser microscopy, the staining was visualized with a confocal laser microscope (Carl Zeiss, LSM 710, Oberkochen, Germany), and the images were processed using the ImageJ or ZEN program.

### Statistical analysis

Statistical analyses were performed using the GraphPad Prism software (version 7.0). The data were presented as mean ± standard error of the mean from at least three independent experiments with similar results. For quantitative analysis of immunoblots, the expression levels of proteins were quantified by densitometry of the bands using Image J. For the quantification of immunofluorescence images, the number of cells used for each representative experiment was indicated. One-way ANOVA was used to calculate *P* values for multiple group analysis. *P* < 0.05 was considered statistically significant.

## Results

### SNX5 and SNX6 selectively regulated the trafficking of CI-MPR and VMAT2

**Figure 1:**
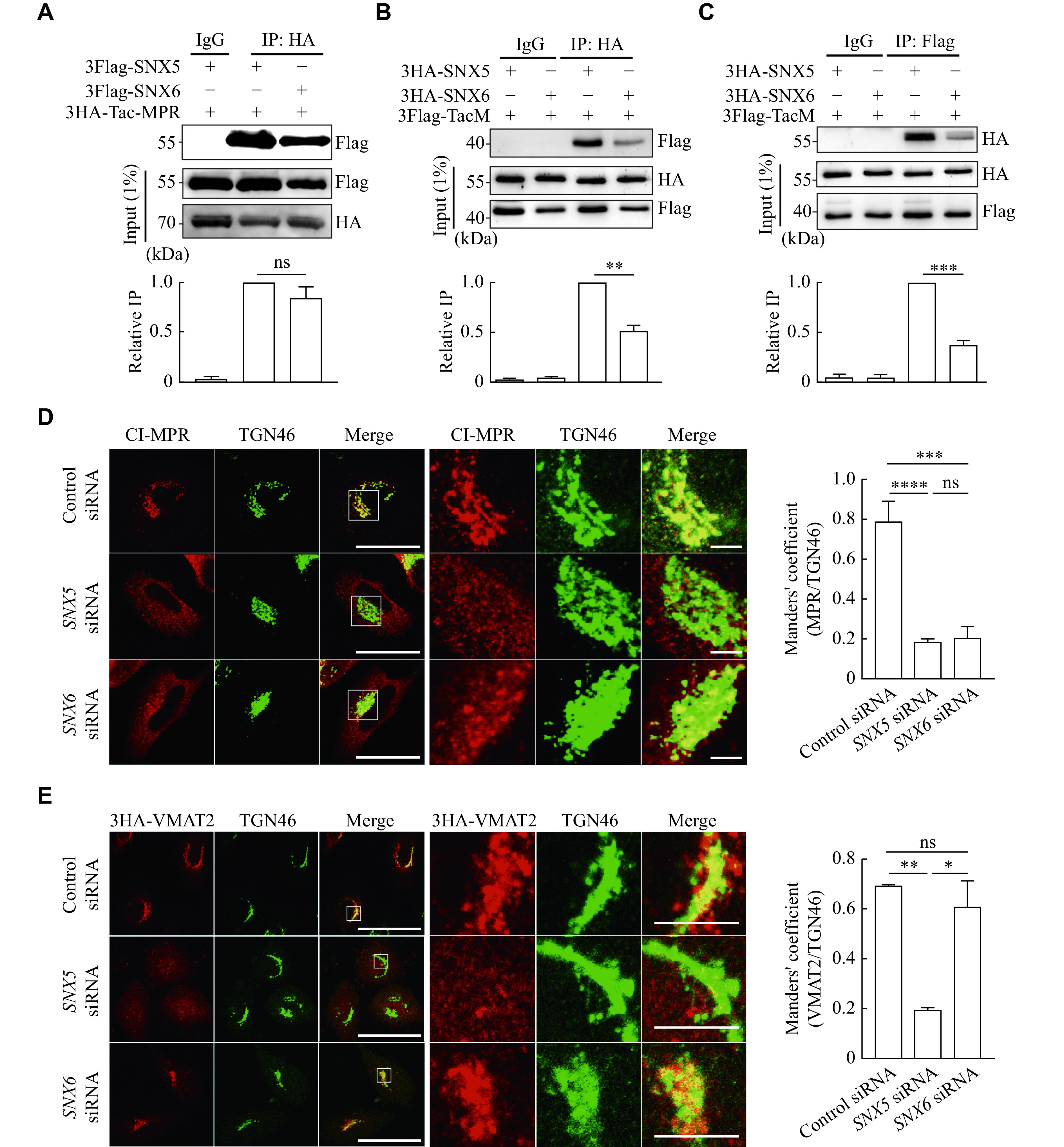
SNX5, but not SNX6, specifically interacted with VMAT2 and regulated the subcellular localization of VMAT2.

Previously, we found that SNX5, but not SNX6, interacted with VMAT2. Although SNX5 and SNX6 share 79% of the residues in the rat protein ( ***Supplementary Fig. 1 ***, available online), they differ in the phosphoinositide binding spectrum, subcellular localization, and binding proteins. To examine the structural determinants essential for their functional differences, we first demonstrated whether SNX5 and SNX6 played distinct roles in regulating retrograde trafficking of two well-characterized cargo proteins, CI-MPR and VMAT2. Because both membrane proteins were characterized for their dependence on the C-terminus for membrane trafficking, we thus used the chimeric proteins generated in our laboratory by fusing the C-terminus of CI-MPR and VMAT2 with the Tac protein (interleukin-2 receptor α-subunit), namely Tac-MPR and TacM (Tac-VMAT2), respectively ^[ 21– 22] ^. In HeLa cells, the overexpressed Flag-tagged SNX5 and SNX6 showed similar binding affinity to the HA-tagged Tac-MPR from the co-immunoprecipitation (co-IP) experiment ( ***Fig. 1A***). However, SNX5, but not SNX6, showed a significantly higher binding affinity to VMAT2 ( ***Supplementary Fig. 2A ***, available online), while immunofluorescent staining showed that SNX5 and SNX6 partially colocalized with VMAT2, indicating the transient interaction ( ***Supplementary Fig. 2B ***). Furthermore, similar results were also achieved by using the chimeric protein TacM, which showed a high affinity in binding to SNX5, but not SNX6 ( ***Fig. 1B*** and ***1C***). To examine the physiological significance of this interaction, we used siRNA-mediated knockdown for *SNX5* or *SNX6* in HeLa cells ( ***Supplementary Fig. 3 ***, available online) and the results showed that the depleted expression of SNX5 and SNX6 altered the subcellular localization of CI-MPR ( ***Fig. 1D***). On the contrary, only the reduced expression of SNX5, but not SNX6, altered subcellular colocalization of VMAT2 with *trans*-Golgi network protein 46 (TGN46; ***Fig. 1E***). These results suggested that SNX5 and SNX6 were involved in different sorting pathways.

**Figure 2:**
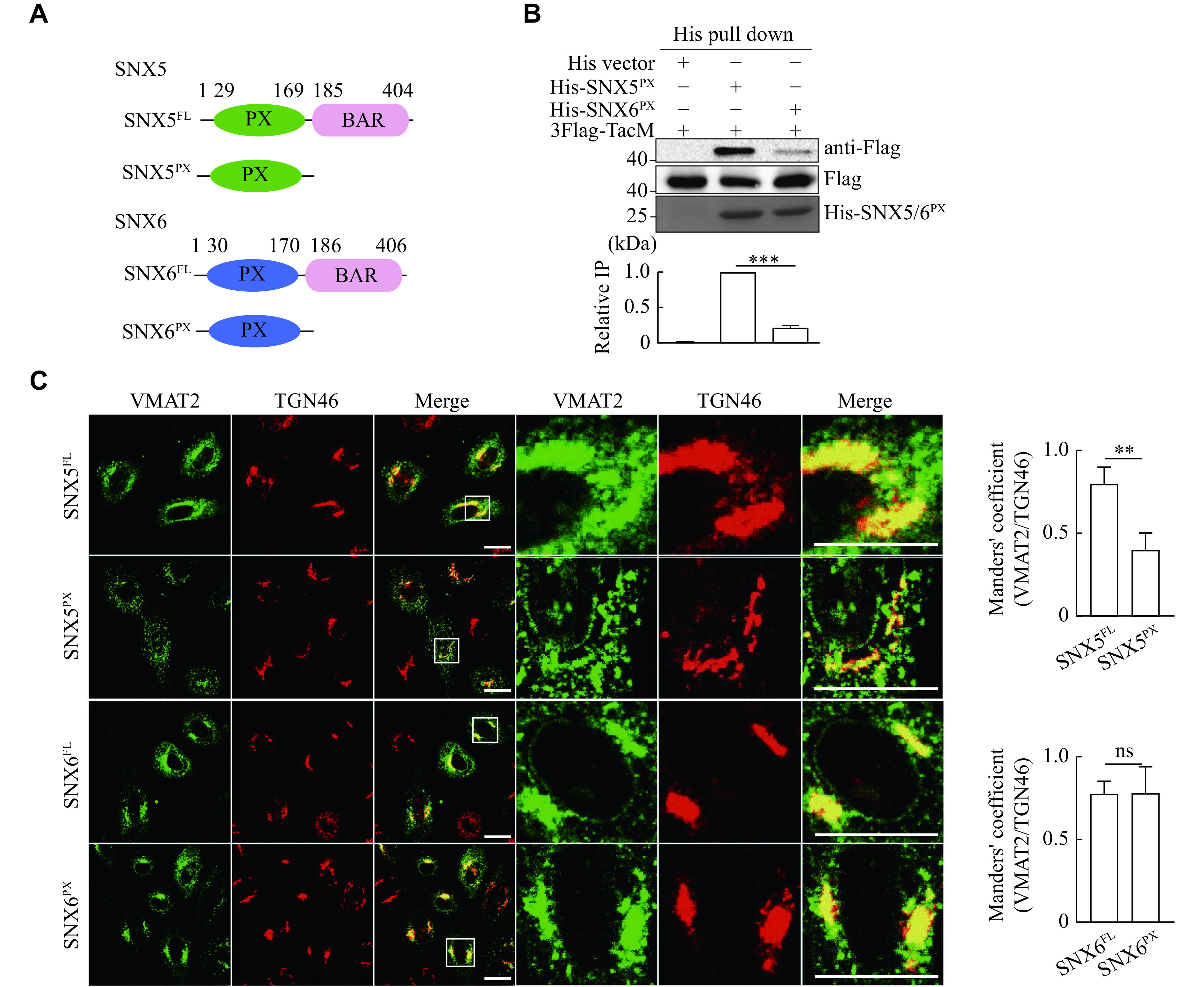
The Phox (PX) domain is required for the interaction of SNX5 and VMAT2.

The N-terminal PX domain of SNX5 or SNX6 was thought to interact with phosphoinositide; but more recently, it has been found to interact directly with proteins ( ***Fig. 2A***) ^[ 23– 24] ^. Interestingly, we have previously shown that the N-terminal PX domain of SNX5 is responsible for its interaction with VMAT2 ^[ 25] ^. Because the PX domain of SNX5 and SNX6 shares a high similarity ( ***Supplementary Fig. 1 ***), we assessed their interactions with TacM using His-tagged recombinant PX domain of SNX5 and SNX6. As shown in ***Fig. 2B***, only His-SNX5 ^PX^, but not SNX6 ^PX^, pulled down TacM from the cell extracts. We also found that the overexpressed PX domain of SNX5, but not SNX6, functionally disrupted the TGN46 colocalization with VMAT2 ( ***Fig. 2C***), strongly indicating that the PX domain of SNX5 and SNX6 may contain structural determinants for their distinct affinity in binding to VMAT2 and regulating its membrane trafficking.

### The PX domains of SNX5 and SNX6 contain distinct residues for protein binding

**Figure 3:**
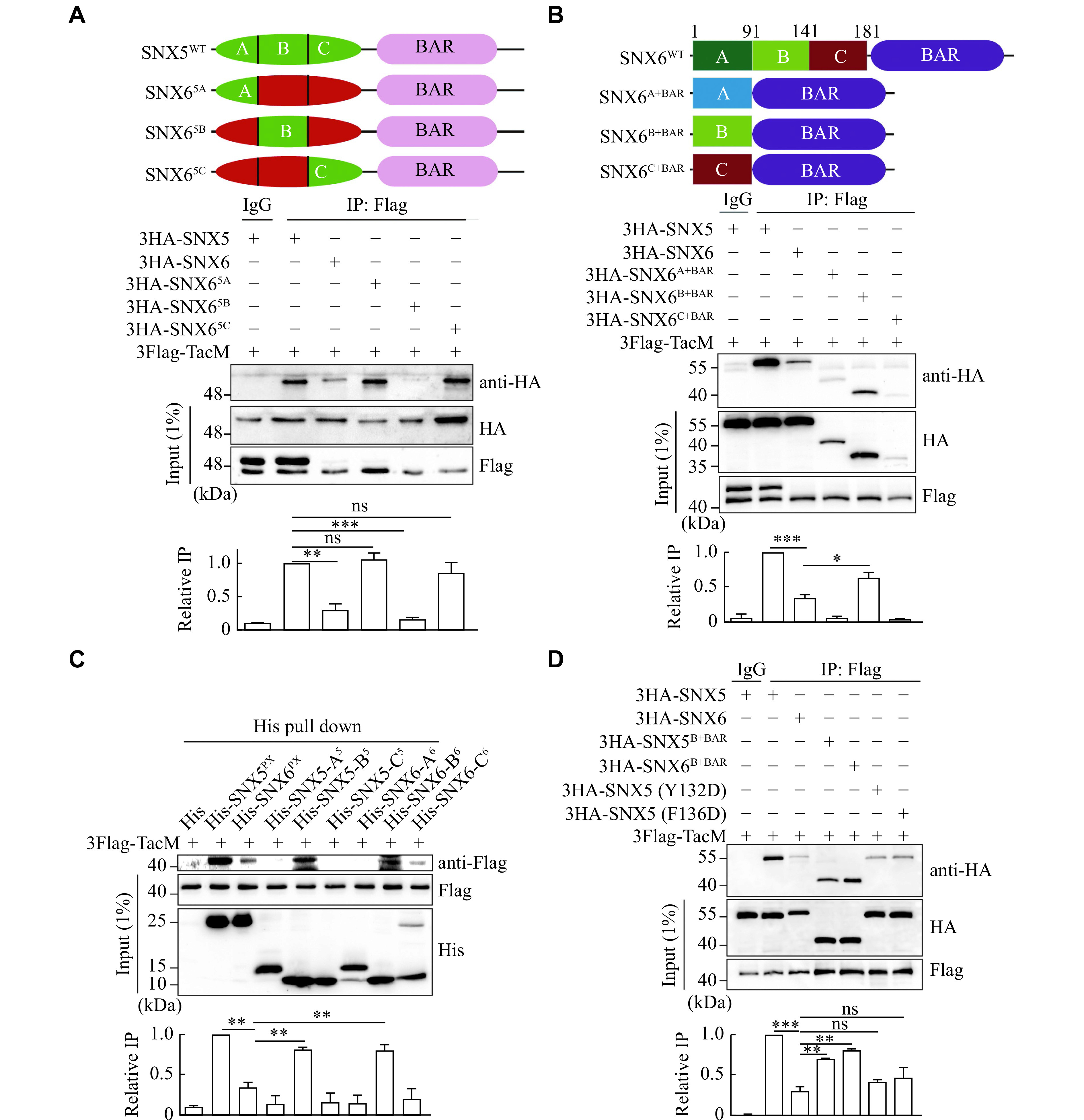
The PX domains of SNX5 and SNX6 contain distinct residues for protein binding.

To determine the key residues of the PX domain of SNX5 or SNX6 essential for recognizing cargos, we used the chimeric mutagenesis approach. Based on the structural features of the PX domain from previous studies and the sequence differences of the PX domain between SNX5 and SNX6 ( ***Supplementary Fig. 1 ***) ^[ 10, 26– 27] ^, we divided their PX domains into three fragments: head group A ^5/6^ (1–90), two-helix loop B ^5/6^ (91–140), and helix linker C ^5/6^ (141–181). We genetically generated the chimeric proteins by swapping these fragments between the two protein PX domains accordingly ( ***Fig. 3A***). Using a co-IP assay to examine the binding affinity of these constructs with TacM, we noticed that replacing A ^6^ or C ^6^ fragments of SNX6 with those of SNX5, respectively (SNX6 ^5A^ and SNX6 ^5c^), significantly restored the binding affinity of the chimeric protein SNX6 with TacM, comparable with that of wild type SNX5 ( ***Fig. 3A***). Notably, the helix loop (SNX6 ^5B^) chimeric protein failed to restore the binding of SNX6 and TacM. These results suggest one of the following two possibilities: the fragments A and C of SNX5, but not SNX6, might contain residues essential for their binding to VMAT2, or the fragment B of both SNX5 and SNX6 might include the binding residue, while the fragment A and C of SNX6 contained inhibitory residues to inhibit the interaction between SNX6 and VMAT2. It is worth mentioning that 3Flag-TacM from the input sample was shown in two bands ( ***Fig. 3A***), but not from the co-IP samples, which may be a result of the binding preference of protein A/G agarose for the lower molecular weight form of the protein. The nature of the two bands of 3Flag-TacM remains unclear, although the Tac protein (IL2RA) has been shown to undergo posttranslational modification by glycosylation.

### The analysis of the special motif within SNX5 required for its interaction with VMAT2

To examine which mechanism is involved in the binding of PX domain to VMAT2, we overexpressed chimeric protein containing each fragment and BAR domain from SNX6 PX domain separately, and found that only the fragment B ^6^ might be able to interact with TacM by co-IP ( ***Fig. 3B***). However, the expression levels of SNX6 ^A+BAR^ and SNX6 ^C+BAR^ were very low, which might be due to their instability or cytotoxicity in cells ^[ 28] ^. To further confirm their binding specificity, we used an *in vitro* pull-down assay by using these individual recombinant protein fragments. As shown in ***Fig. 3C***, only fragment B of both SNX5 and SNX6 specifically pulled down TacM. In particular, we tested two point mutations at the Y132D and F136D sites in the B ^5^ fragment, which were reported to be required for the specific binding between SNX5 and CI-MPR ^[ 23] ^. As shown in ***Fig. 3D***, these mutants led to the deprivation of their interaction with TacM, further validating the specificity and importance of the B fragment for the recognition and binding of cargo proteins.

Taken together, these results strongly supported our second hypothesis that the B fragment of both SNX5 and SNX6 had a special binding affinity for VMAT2, which was inhibited, in SNX6, by the A and C fragments. Thus, these two fragments could contain specific inhibitory residues in SNX6 to block the interaction of its PX domain with VMAT2.

### Identification of key residues in SNX6 required to inhibit its interaction with VMAT2

**Figure 4:**
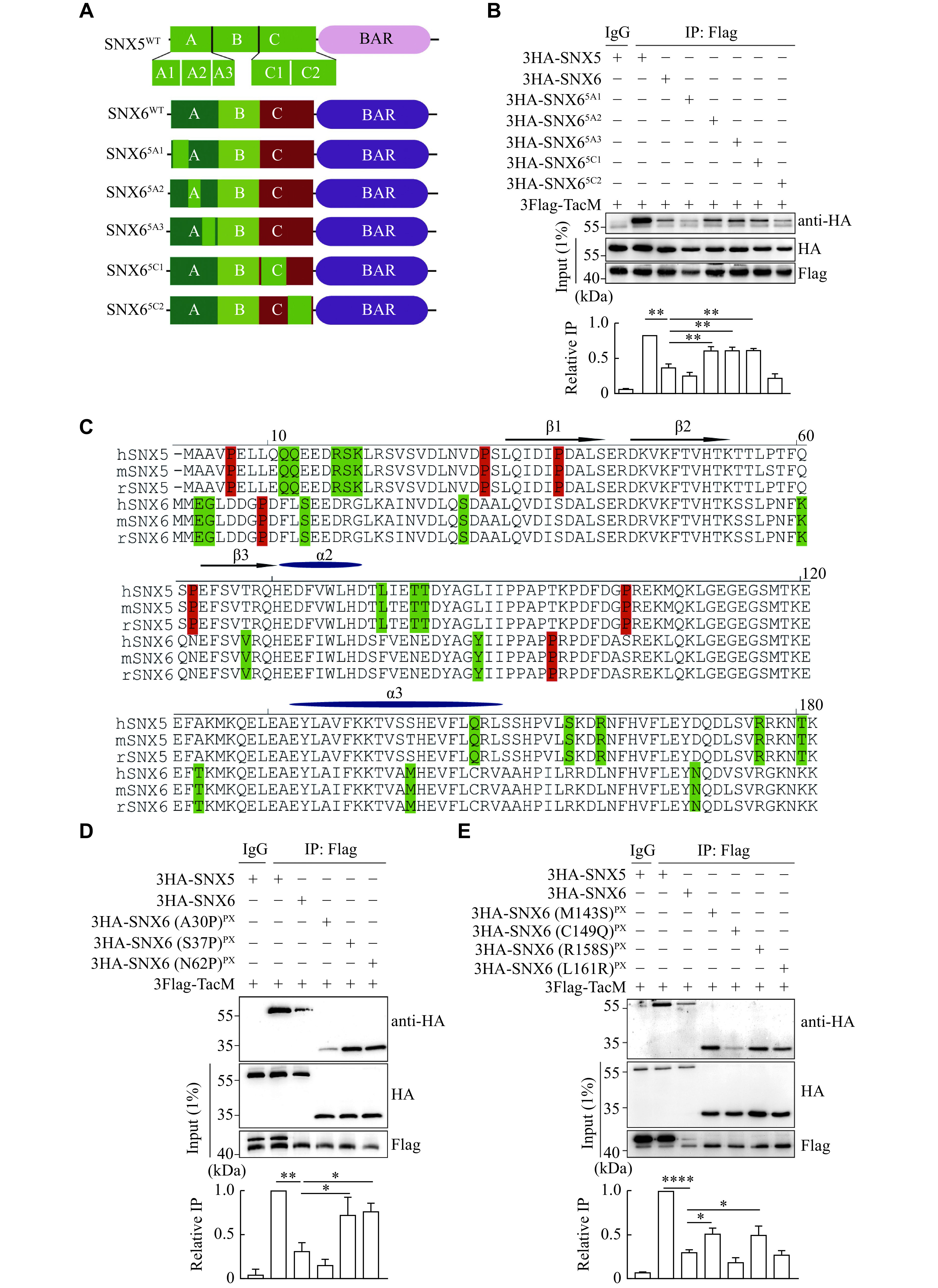
Identification of key residues in SNX6 required for inhibiting its interaction with VMAT2.

To screen for the region and residues in the A and C fragments of the SNX6 PX domain that are responsible for inhibition, we further divided these fragments into five subregions to generate chimeric proteins. The A fragment was divided into three subregions as A1, A2, and A3 with the corresponding amino acid sequences as 1–35, 36–57, and 58–90, respectively, and the C fragment into two subregions as C1 and C2 with the corresponding amino acid sequences as 141–160 and 161–181 ( ***Fig. 4A***), respectively. The corresponding subregions of SNX6 were replaced by those of SNX5 for a set of chimeric proteins to examine their binding affinity to TacM using the co-IP assay. As shown in ***Fig. 4B***, compared with SNX6 ^WT^, chimeric proteins tagged with HA, SNX6 ^5A2^, SNX6 ^5A3^, and SNX6 ^5C1^, showed partial binding to TacM, suggesting that these three subregions in SNX5, altered in SNX6, contained the key motifs required for specific binding to VMAT2. Therefore, based on the differences in the amino acid sequence of these three subregions between SNX5 and SNX6 and the structural significance of prolines in the PX domain, we focused on mutagenizing amino acids in SNX6 corresponding to those in SNX5 at the A30, S37, N62, M143, C149, R158, and L161 sites ( ***Fig. 4C***). As shown in ***Fig. 4D*** and ***4E***, mutants with point mutations of S37P, N62P, M143S, R158S, and L161R of SNX6 clearly showed a restoration of their binding to TacM, compared with that of SNX6 ^WT^, suggesting that these four residues in SNX5 played a critical role in the specific interaction with VMAT2. On the contrary, the altered residue sequence for these four amino acids in SNX6 may be responsible for the altered structure of these subregions required for the interaction. Furthermore, these results support the idea that SNX5 and SNX6 may play different regulatory functions in retrograde membrane trafficking of VMAT2.

**Figure 5:**
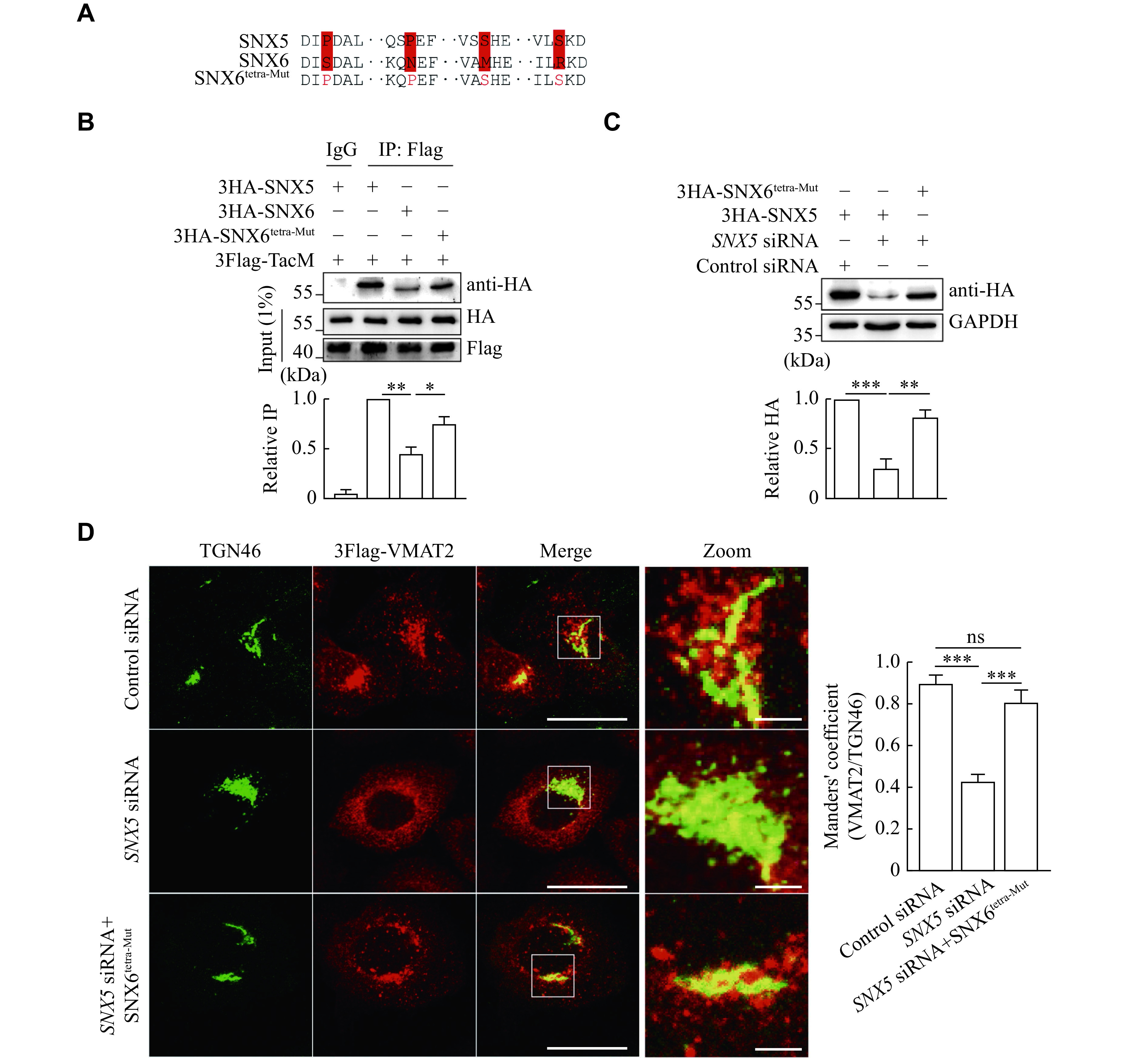
Overexpression of SNX6 ^tetra-Mut^ restored its regulatory function in TGN targeting of VMAT2.

To test whether these key residues are functionally critical for SNX5 but not for SNX6 in regulating the membrane trafficking of VMAT2, we first generated a tetramutant SNX6 construct (SNX6 ^tetra-Mut^), in which the following residues, S37, N62, M143, and R158, were mutated to S37P, N62P, M143S, and R158S in the corresponding amino acid for SNX5 ( ***Fig. 5A***). As shown in ***Fig. 5B***, the SNX6 tetramutant showed a strong interaction with TacM, compared with wild-type SNX6. Furthermore, we made several silent nucleotide mutations to allow this construct to be resistant to specific *SNX5* siRNA ( ***Fig. 5C***). Consistent with the previous results, the loss of SNX5 expression induced by siRNA-mediated knockdown altered the subcellular localization of VMAT2 away from TGN. However, overexpressed SNX6 ^tetra-Mut^ completely rescued the mistargeting of VMAT2, presumably by restoring the retrograde trafficking of the transporter ( ***Fig. 5D***).

### Key residues in SNX5-PX essential for regulating the large dense core vesicles (LDCVs) targeting of VMAT2 in PC12 cells

**Figure 6:**
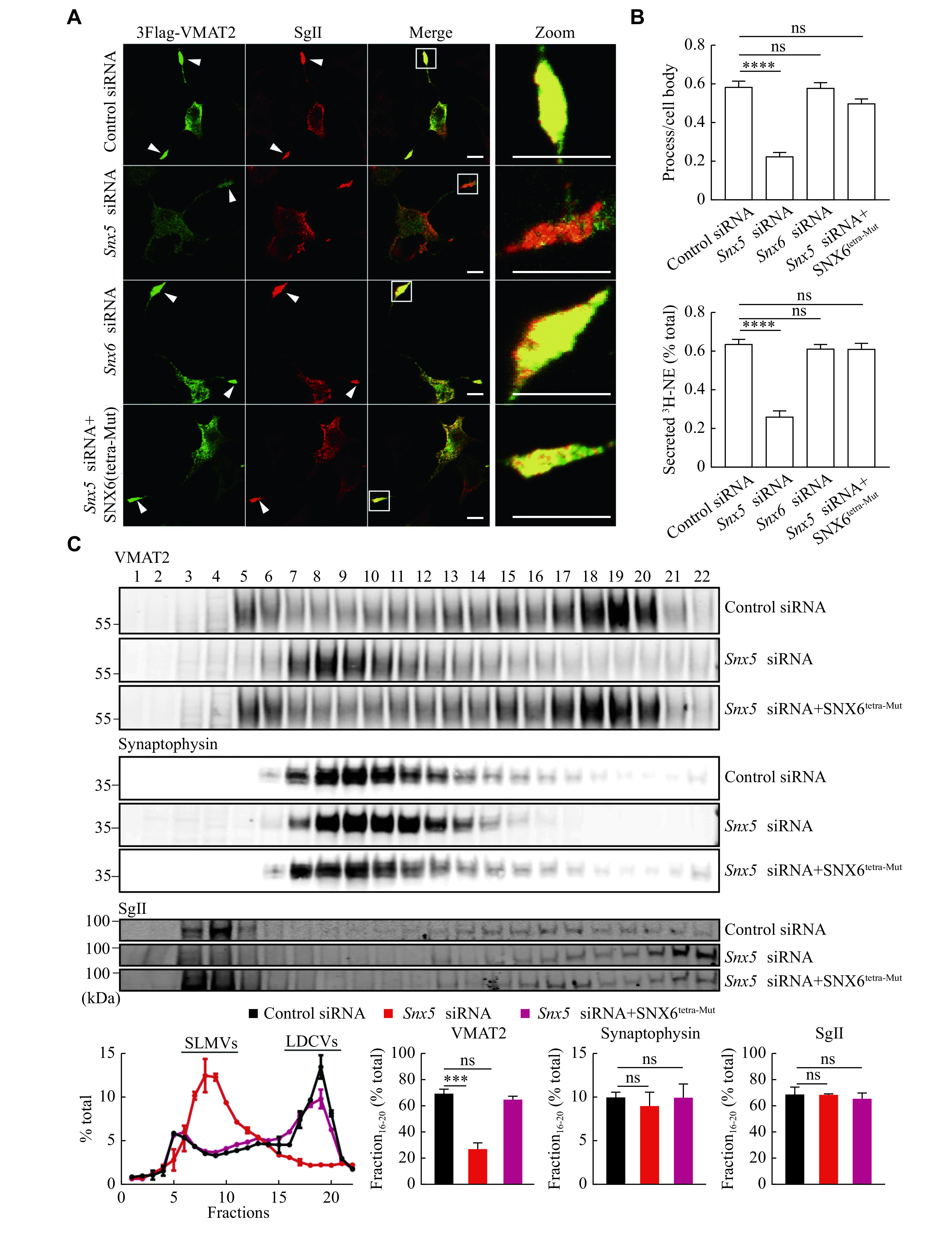
Overexpression of SNX6 ^tetra-Mut^ restored the LDCVs targeting and function of VMAT2 in PC12 cells.

We then examined the functional relevance of the key residues of SNX6 (Ser ^37^, Asp ^62^, Met ^143^ and Arg ^158^), identified in the mutagenesis study in VMAT2 targeting to LDCVs using PC12 cells. PC12 stable transformants expressing the Flag-tagged VMAT2 were first studied, upon siRNA-mediated knockdown of endogenous *Snx5*, and it showed a disrupted colocalization of VMAT2 with SgⅡ, the marker for LDCVs, at the tips of neural processes ( ***Fig. 6A*** and ***Supplementary Fig. 4*** [available online]). However, the loss of *Snx6* expression had no effect on the LDCV targeting of VMAT2. Consistently with its biochemical results, overexpressed SNX6 ^tetra-Mut^ clearly restored vesicular targeting of VMAT2 to LDCVs, which was altered in control cells after *Snx5* knockdown ( ***Fig. 6A*** and ***Supplementary Fig. 4 ***), strongly supporting the notion that these key residues in SNX5 were important for structural-dependent protein interaction and function. Furthermore, the vesicular localization of VMAT2 to LDCVs, required for its transport activity, was confirmed by the pharmacological analysis. As shown in ***Fig. 6B***, the depleted expression of endogenous SNX5, but not SNX6, significantly decreased the depolarization-dependent release of preloaded ^3^H-norepinephrine ( ^3^H-NE) from PC12. Since NE is one of the monoamine transmitter substrates for VMAT2 and normally is packaged inside of both synaptic vesicles and LDCVs in neurons, mostly in LDCVs in PC12 cells, this result indicated that the altered VMAT2 membrane targeted to non-secretory subcellular membrane compartments. Consistently, SNX6 ^tetra-Mut^ restored the stimulated release of ^3^H-NE in *Snx5* knockdown cells, suggesting that the SNX5-like SNX6 ^tetra-Mut^ functionally restored the LDCV targeting of VMAT2.

The effect of SNX6 ^tetra-Mut^ on the subcellular membrane trafficking of VMAT2 was further examined biochemically by using the density gradient fractionation of PC12 transformants. As shown in ***Fig. 6C***, HA-VMAT2 was identified in fractions containing the LDCV marker SgⅡ in heavy fractions in control cells, but it showed an altered distribution to light fractions after *Snx5* knockdown, suggesting the comigration of two proteins in LDCV. Similarly, overexpressed SNX6 ^tetra-Mut^ restored the LDCV targeting of VMAT2 due to the loss of SNX5. In conclusion, both immunofluorescent staining and gradient fractionation analysis provided biochemical and cellular evidence that residues, such as Pro ^36^, Pro ^61^, Ser ^142^ and Ser ^157^ in the PX domain of SNX5, were structural determinants not only for their interactions with VMAT2 but also for the functional regulation of vesicular targeting *via* retrograde trafficking of the transport protein to LDCVs for the secretory process in both endocrine glands and monoamine neurons. Without these residues, SNX6 no longer occupied the structure of PX domain essential for its interaction with VMAT2 and the functional regulatory role in the targeting of VMAT2 to LDCVs.

## Discussion

In the current study, we systematically investigated whether the distinct regulatory role of retromer complex subunits SNX5 and SNX6 in the membrane trafficking of VMAT2 is determined by their structural differences using a set of molecular and cellular approaches. We first confirmed the requirement and sufficiency of the PX domain of SNX5 for its specific interaction with VMAT2, and identified the two key residues within this domain, Y132 and F136, were critical for its double-helix structures. Then, we used point mutagenesis methods and identified that the middle fragments of both SNX5 and SNX6 contained a sequence for interaction with VMAT2. However, the sequences flanking the middle fragment in SNX6 may contain structural determinants that alter the protein structure required for its interaction with VMAT2. Furthermore, we identified four residues within these regions of SNX5, such as Ser ^37^, Asp ^62^, Met ^143^ and Arg ^158^, for their essential roles in the interaction with and functional regulation of VMAT2 in its retrograde trafficking and LDCV targeting. Importantly, SNX6 ^tetra-Mut^ with altered residues mimicked that in SNX5 was shown to have a similar function of SNX5 in protein interaction and regulatory function in VMAT2 membrane trafficking.

We previously reported that the key component of retromer, Vps35, interacted with VMAT2 using cell based *in vivo* biochemical analysis ^[ 29] ^. We also showed that the depletion of VPS35 disturbed VMAT2 subcellular localization at the TGN and decreased its protein stability, although whether this interaction depended on other retromer components or not was not clear. Importantly, we recently reported direct interaction between SNX5 and VMAT2, suggesting a unique role of SNX5 in the retrograde trafficking of VMAT2 ^[ 25] ^. Interestingly, VPS35 has long been considered the sorting protein for cargo in the retrograde transport pathway, as it recognizes and binds to cargo proteins. On the other hand, numerous studies strongly support a vital role of the sorting nexin proteins in the recognition and transport of cargo proteins ^[ 24, 30– 32] ^. One such study has shown that SNX-BAR domains are correlated with the cytoplasmic tail of CI-MPR and mediate its trafficking ^[ 5– 6] ^. However, other studies indicate that the binding between SNXs and CI-M6PR or IGF1R is mediated by the PX domain of SNX5 or SNX6 and a bipartite motif termed SNX-BAR-binding motif (SBM) in the cargo proteins ^[ 22] ^. Although we have shown that both SNX5 and SNX6 interact with CI-M6PR, their interactions with VMAT2 are significantly different, supported not only by the biochemical study but also by functional cell biology data in the current work. With the two SNXs are generally considered interchangeable isoforms, their differences in lipid binding, protein interactions and function have been widely reported without structural and mechanistic studies. It is worth noting that the recombinant SNX6-PX domain was extremely difficult to produce during the current study, compared with SNX5 in our structural analysis in previously reported work, consistent with other literature reports ^[ 28, 33] ^, suggesting that there may be structural differences between SNX5 and SNX6.

Our recent structural study showed that two additional α-helices and the unique close double PXXP motif inserted into the SNX5 PX domain, compared with other sorting nexin proteins, which may be correlated with the loss of its binding pocket site with PI3P instead of PI(4,5)P2 ^[ 10] ^. This finding provides a molecular basis for its binding to other membrane proteins, demontrating a dual role of the PX domain that is unique among a handful of proteins. Among the SNX family, only three proteins (5, 6, and 32) contain the PXXPXXP motif, followed by a short thirteen amino acid sequence that is not similar to other members of the family. The chimeric and deletion mutagenesis study on SNX5 and SNX6 supported our hypothesis that the middle part of SNX6 (fragment B in ***Fig. 3A*** and ***3B***, amino acid sequence of 91–140) contained the double PXXPXXP motif for their specific binding to VMAT2. Our data also indicated that both fragements A and C of SNX6 did not bind to the transporter; instead, they had an inhibitory effect on fragment B of SNX6. Thus we focused on the residues that differed in these two fragments between the two proteins with structural relevance, such as proline and serine. The results from the point mutagenesis analysis supported that these residues determined the structure of SNX6 different from that of SNX5, thus providing the first experimental evidence to support a molecular mechanism underlying their known differences in lipid binding, protein interactions, and functional role in membrane trafficking.

Vesicular trafficking of VMAT2 is involved in its targeting to LDCVs at the site of TGN and retrograde endosome-to-TGN trafficking after exocytosis of the secretory vesicles at the plasma membrane. Currently, the unique feature of SNX5 in its binding to PI(4,5)P _2_ and the only SNX on synaptic vesicles suggest its role in cargo cognition at the site of early endosomes ^[ 10, 15] ^. On the other hand, although SNX6 does not interact directly with VMAT2, it is unclear whether it participates in membrane trafficking indirectly by regulating the formation of the retromer complex or other sorting machinery. The lipid binding of SNX6 to PI(4)P indicates a potential role at the site of TGN for targeting cargo proteins. Moreover, SNX6 interacts with p150 ^Glued^ to ensure the transport of cargo proteins along the microtubules ^[ 12– 13] ^. The subsequent combination of SNX6 with PI(4)P at the TGN promotes the separation of the cargo and p150 ^Glued^ in the TGN, ensuring that the cargo protein is accurately unloaded at the destination. Because the membrane trafficking of CI-MPR and VMAT2 are both TGN localized proteins in an immunostaining pattern but differ in their membrane association, with the former involved in TGN-endosome recycling and the latter in LDCV-plasma membrane-TGN recycling, the different roles of SNX5/SNX6 in their retrograde trafficking may further indicate the importance of SNX6 in the TGN targeting of VMAT2 to the secretory vesicles. Thus, understanding how SNX6 is involved in the assembly of cytosolic machinery during the formation of LDCVs and targeting of cargo proteins, such as VMAT2, is critical to unveil the relevance of the structural determinants identified in the current study. One such experimental approach would be to examine whether the coexistence of SNX6 interacting proteins or/and PI(4)P could alter its binding affinity to VMAT2 *in vitro* and *in vivo*. Furthermore, a structural analysis of the difference between SNX5 and SNX6 with a focus on the residues identified in this work may provide a much better resolution of how these two isoforms functionally vary in retrograde membrane trafficking of signaling membrane proteins in cells.

Taking advantage of the sequence difference between SNX5 and SNX6, we have identified four critical residues (Pro ^36^, Pro ^61^, Ser ^142^, and Ser ^157^) in SNX5 for its interaction with VMAT2. The mutations of these corresponding residues in SNX6 (Ser ^37^, Asp ^62^, Met ^143^ and Arg ^158^) can restore its binding affinity to VMAT2. To understand how these residues play a role in the structural requirement of the interaction, we accessed structural data from the Alphafold2 AI analysis program for rat SNX5 ( https://alphafold.ebi.ac.uk/entry/B1H267) and rat SNX6 ( https://alphafold.ebi.ac.uk/entry/B5DEY8), respectively. As shown in ***Supplementary Fig. 5*** (available online), the tertiary structures of the two SNXs were very similar. Fragment B of SNX5 and SNX6 (91–141) started with the PXXPXXP domain for α-2 helix with an open space for easy access of the interacting proteins (S5B). Interestingly, these four residues are distributed in the vicinity domains that form a binding pocket (S5B). Specifically, SNX5-P36 and P61 are the structural turning points that flank the β-1 and β-2 sheets. However, SNX6-S37 seems to be part of the extended β-2 sheet in SNX6 (S5C). Additionally, the side chains of SNX6-M143 and R158 appear to be more interactive with the neighboring residues from other structural domains, suggesting a role for their structural differences between the two SNXs. Nevertheless, this AI-assisted structural analysis is still in its early stage, and future experimental studies on the high-resolution structure of SNX6 would be more helpful in understanding its role in cargo trafficking.
